# Minimally Manipulative Method for the Expansion of Human Bone Marrow Mesenchymal Stem Cells to Treat Osseous Defects

**DOI:** 10.3390/ijms20030612

**Published:** 2019-01-31

**Authors:** Logan M. Lawrence, Andrew Cottrill, Amrita Valluri, Gaetano Marenzi, Krista L. Denning, Jagan Valluri, Pier Paolo Claudio, James B. Day

**Affiliations:** 1 Cabell Huntington Hospital Laboratory, Department of Pathology, Marshall University Joan C. Edwards School of Medicine, Huntington, WV 25705, USA; loganlawrence38@gmail.com (L.M.L.); haught5@marshall.edu (K.L.D.); 2 Department of Biological Sciences, Marshall University, Huntington, WV 25705, USA; cottrill40@live.marshall.edu (A.C.); valluri3@live.marshall.edu (A.V.); valluri@marshall.edu (J.V.); 3 Department of Neuroscience, Reproductive, and Odontostomatologic Sciences, University of Naples “Federico II” 80131, Italy; gaetano.marenzi@gmail.com; 4 Department of BioMolecular Sciences, National Center for Natural Product Research, University of Mississippi, University, MS 39216, USA; 5 Department of Orthopaedic Surgery, Marshall University Joan C. Edwards School of Medicine, Huntington, WV 25705, USA

**Keywords:** bone marrow mesenchymal stem cells, autologous transplant, regenerative medicine, fracture healing, bone defects, traumatic injuries

## Abstract

Lack of standardization of clinically compliant culture protocols of mesenchymal stem cells for re-implantation in humans have hindered clinical progress in the field of tissue regeneration to repair maxillofacial and orthopedic defects. The goal of this study was to establish a clinically relevant osteogenic protocol for collection and expansion of autologous stem cells to be used at Marshall University for re-implantation and repair of maxillofacial and orthopedic conditions. Human bone marrow (hBM) samples were collected from patients undergoing intramedullary nail fixation for closed femoral fractures. hBM mesenchymal cells were expanded by growing them first in Petri dishes for two weeks, followed by a week of culture using Perfecta 3D Hanging Drop Plates^®^. Various scaffold materials were tested and analyzed for cellular integration, vitality, and differentiation capacity of harvested hBM-MSCs including: 60/40 blend of hydroxyapatite biomatrix; Acellular bone composite discs; Allowash^®^, cancellous bone cubes; PLGA (poly lactic-co-glycolic acid); and Woven chitin derived fiber. We found that the 3D spheroid culture allowed production of hBM mesenchymal cells that retained osteoblast differentiation capacity over a monolayer culture of hBM-MSCs without the need to use chemical or hormonal modulation. We also observed that hydroxyapatite and Allowash cancellous bone scaffolds allowed better cell integration and viability properties as compared to other materials tested in this study. In conclusion, the multimodal culture methodology we developed creates actively differentiating stem-cell spheroids that can then be readily utilized in clinical practices to improve the regeneration of tissues of the head and the body.

## 1. Introduction

The field of regenerative medicine is rapidly evolving and although stem cell technologies have been exhaustively researched in the laboratory setting, they have not yet been widely integrated into the clinical setting. This is largely due to a general lack of traditional clinical utility and standardization that is necessary to implement these complex therapies in both a regulatory compliant and consistent manner. One of the most widely studied stem cell applications is that of using adult multipotent mesenchymal stem cells to assist in a variety of wound healing practices [1]. We present a novel, clinically relevant osteogenic protocol for autologous mesenchymal stem cell expansion and re-implantation.

Mesenchymal stem cells (MSCs) are renowned for their differentiation potential and versatility in regenerative medicine for the treatment of a breadth of issues ranging from chronic diseases to traumatic injury [2]. Bone marrow-derived MSCs (hBM-MSCs) are useful in healing traumatic bone injuries by inducing osteo-regeneration. 5–10% of bone fractures result in a “non-union” clinical outcome wherein the patient’s bone fails to heal itself from injury [3]-by introducing actively differentiating MSC’s and thus reversing the process. More promising, such a developed technique could be adapted to large osseous defects (even coupled to scaffolding techniques) and provide an advanced means for autologous bone regeneration resulting from trauma, infections, and even tumor resections. MSC therapies are not limited only to non-union fractures and could be used in treating a variety of other traumatic or degenerative orthopaedic or maxillofacial illnesses such as bone defects and cartilage replacement [2]. Use of MSCs has been focused most heavily on soft tissue injury and immune disorders, but their utility has been implicated for the treatment of a variety of other conditions.

In order to develop a clinical practice using MSCs, expansion of MSC populations in vitro is critical to achieving a therapeutically relevant number of active cells [4]. In cases where the body cannot heal itself via natural stem cell recruiting pathways the cause is often a defect in stem cell induction and signaling pathways. In older individuals this may occur due to decreased overall nutritional and growth factor deficits in the native stem cell environments. This natural aging process has a direct impact on bone regenerative potential and MSCs decrease in both number and activity in the body [5].

Cell-based therapies also often require the use of a biomatrix, also referred to as scaffold, to hold the implanted cells in the intended local environment within the body at the site of injury [6]. In this study widely available and FDA approved materials, including 60%/40% (matrix to pore space ratio) hydroxyapatite blends, cancellous bone cubes, patient bone fragments prepared in an acellular manner, PLGA poly(lactic-co-glycolic acid) constructs, and woven chitin bio fibers were assessed for their compatibility with the expanded MSC populations.

MSC auto-transplantation protocols could be used to dramatically improve the outcomes of serious bony defects and non-unions. However, the limiting factor of any such treatment option is the time needed to expand an individual’s extracted stem-cell population [7]. Human bone marrow mesenchymal stem cells (hBM-MSCs) can be cultured using a variety of different techniques and methodologies, including traditional monolayer culture, biomatrix embedding of cells, and as of recently, self-assembled spheroid culture [8]. Spheroid culture in vitro has been shown to exhibit significant advantages over other traditional culture modalities regarding osteogenic potential, cell-to-cell contact, up-regulation of osteogenic genes and proteins, and calcium auto-deposition without the utilization of exogenous osteoblast inducer reagents (OIR) [9].

The protocol we developed cultures MSCs in vitro within a 3-week window. We combined two methodologies: A traditional, two-dimensional substrate-based adherent monolayer method and a three-dimensional spheroid culture method.

It is known that the ability of stem cells to form clonal colonies while maintaining multipotent differentiation potential declines over successive expansions in a two-dimensional environment [10]. This is largely due to the polar nature of the monolayer itself. The layer of cells forms on the bottom of the plate and expands along a single plane. The polar, flat, nature of the cell population causes a differential in media and nutrient exposure, thus increasing the possibility for heterogeneity within the population of cells [11]. Another contributing drawback to monolayer culture is that cell-to-cell surface contact is minimalized and therefore it keeps the cell population from being representative of the in vivo behavior of MSCs in the body. This lack of cell signaling presents the need for exogenous growth and differentiation factors in maintaining biologically active MSC populations in vitro, further removing the population from an accurate representation of MSCs within the body.

To better simulate in-vivo conditions, three-dimensional (3-D) cell culture platforms have become a major focus in regenerative medicine in recent years. Culturing stem-cells in a 3-D environment where cell-cell contact is maximized has been shown to drastically improve the retention of “stemness” qualities in MSCs [12]. This retained differentiation potential is vital in developing any effective stem cell-based therapy, as re-implanting cells that have lost their functional ability to heal wounds would be a futile endeavor.

## 2. Results

### 2.1. Human Bone Marrow (hBM) Sample Collection

Human bone marrow samples were obtained from four patients undergoing intramedullary nail fixation for closed femoral fractures. Patients were selected based on their injuries and were enrolled in an IRB protocol at Marshall University.

hBM samples were collected using either the reamer irrigator aspirator (RIA) and filtration system for BM sample collection or the standard reaming techniques used to prepare the canal for intramedullary implants. Waste blood and serum were collected with a bulb syringe or regular syringe through the nailing portal immediately after reaming. Using these two techniques, mesenchymal stem cells found in both the aspirated material and the collected bony fragments from the reaming were collected.

We found that the RIA method of reaming is less optimal for the collection of viable stem cells. In fact, better proliferative characteristics were observed from the “non-washed” standard reaming collection even though the amount of material collected by this method is often limited.

### 2.2. MSC Characterization

Flow Cytometry analysis was used to characterize the primary cell populations derived from patient donors in this study. Cell markers examined in this study were targeted to the mesenchymal lineage of the native stem cell population present in adult human bone marrow. Table 1 displays the markers that were assessed. The isolated cells were adherent to plastic and displayed fibroblast morphology and surface markers, such as cluster of differentiation 44, 29, 105 and 73 (CD44, CD29, CD105, and CD73), similarly to preparations of MSCs described by other groups derived from bone marrow (BM) [13,14].

**Figure 1:**
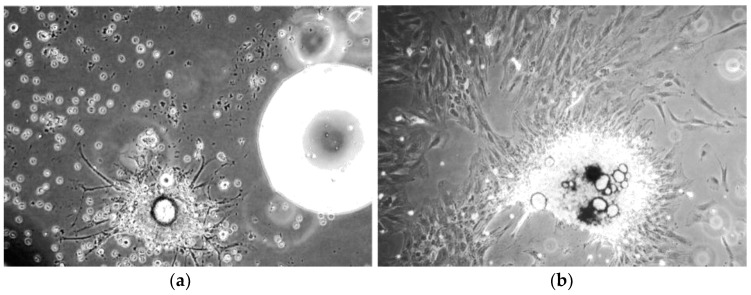
Inverted light microscope images of human bone marrow mesenchymal stem cells (hBM-MSC). (**a**) hBM-MSCs cultured from subject BM004 that were grown in 2D culture at 1 week post-harvest (100× magnification); (**b**) hBM-MSCs cultured from subject BM004 that were grown in 2D culture at 3 weeks post-harvest (100× magnification).

The patient derived cell populations were also imaged on a regular basis to document their growth rate and monitor for unexpected morphological changes. We found that the average in-vitro doubling time of BM001 was 36 h, of BM002 was 120 h, and of BM004 was 23 h. Figure 1 (a and b) show images taken at weeks 1 and 3 of the hBM-MSCs bi-dimensional expansion, respectively [15].

### 2.3. Patient Derived Serum Supplementation Efficacy

**Figure 2:**
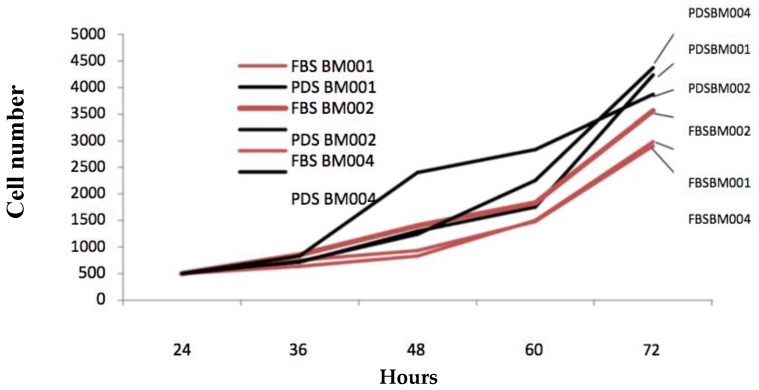
Comparison of hBM-MSCs growth using media supplemented with patient-derived serum (PDS) vs. fetal bovine serum (FBS). The diagram represents grow curves (cell number over time) of hBM-MSC (BM001, BM002, and BM004) grown using medium supplemented with FBS Compared to PDS over 72 h.

One of the novel and keystone aspects of our stem cell expansion protocol is the use of patient derived growth factors in place of the industry standard animal derived growth factors. For virtually all-mainstream cell culture protocols, fetal bovine serum (FBS) products are added to the basal culture medium in order to facilitate cell proliferation [15]. This approach of employing animal based products, although effective, does not meet current FDA regulations. The described protocol substitutes FBS with a supply of growth factors that are directly derived from the patient whose cells are being expanded for therapeutic purposes. This serum, herein referred to as patient derived serum (PDS), was found to not only be a fit substitute, but was mildly more effective at promoting the in-vitro expansion of primary mesenchymal cell populations under the conditions used in this protocol. The patient derived stem cell populations were cultured using media formulations employing either FBS or PDS and assessed for colony population at regular 24-h intervals for 5 consecutive days. The results are displayed in Figure 2 and Table 2. We observed that the hBM-MSC cultured using the patient own derived serum grew faster in three different replica experiments (8.4; 7.7; and 8.7 fold increase) than those cultured with fetal bovine serum (5.8; 7.1; and 5.9 fold increase) with a calculated 6.3 vs. 8.3 cumulative average fold increase, respectively (*p* ≤ 0.05).

### 2.4. Dexamethasone-Induced Differentiation Results

**Figure 3:**
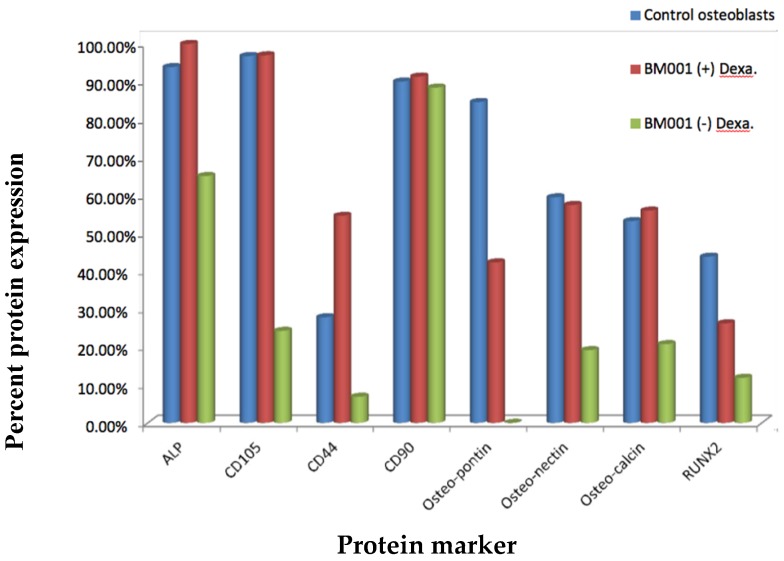
Expression of differentiation markers in hBM-MSC following dexamethasone treatment as measured by Flow cytometer. The histogram is a representative example of the percentages of expression of various bone marrow stem cell and osteoblast differentiation markers, before and after treatment with dexamethasone. Control Osteoblasts were used as positive control. Control osteoblast and BM001 isolated hBM-MSC cells were incubated with anti ALP, CD105, CD44, CD90, Osteopontin, Osteonectin, Osteocalcin, and RUNX2 specific fluoresceinated antibodies and flow cytometry analysis was used to determine percent of expression of the different proteins before and after incubation with dexamethasone to study their differentiation capabilities.

The ability to differentiate in vitro of the primary mesenchymal stem cell populations isolated was verified using a hormonal modulation protocol utilizing dexamethasone, a steroid hormone [16]. The primary cell population of hBM-MSC (BM001) was subjected to a 25-day exposure to dexamethasone followed by flow cytometer assay of various cellular markers (ALP, CD105, CD44, CD90, Osteopontin, Osteonectin, Osteocalcein, and RUNX2). A commercially available cell line of osteoblasts (HOB, Promocell, cat#C-12720) and unstimulated BM001 patient derived BM cells were used as controls. We observed that the primary hBM-MSC cells (BM001) differentiated into osteoblasts following a 25-day treatment with dexamethasone as demonstrated by flow cytometry assay. Figure 3 and Table 3 show that the markers displayed by the treated cells parallel those of known osteoblast cells and are significantly changed with regards to their original naïve states. Treatment with dexamethasone increased expression of ALP, Osteopontin, Osteonectin, and Osteocalcin, in the BM001 cells indicating the activation of differentiation toward the osteoblast lineage.

Further evidence of osteoblast differentiation was the morphological change associated with dexamethasone treatment.

**Figure 4:**
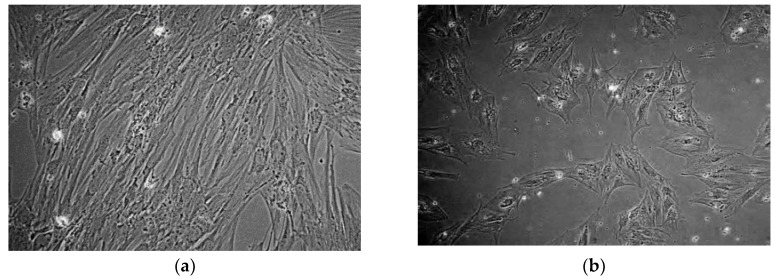
Phase contrast microscopy images of hBM-MSCs before and after dexamethasone treatment. (**a**) BM002 hBM-MSCs grown in the absence of differentiation stimuli, display spindle-shaped cells that formed densely populated colonies (100× magnification); (**b**) BM002 hBM-MSCs grown in the presence of differentiation inducing dexamethasone, display polygonal cells with less densely packed colonies and enlarged cytoplasmic space (100× magnification).

Characteristics of the morphological change included the transition from spindle-shaped cells that formed densely populated colonies (Figure 4a) to polygonal cells with less densely packed colonies and enlarged cytoplasmic space (Figure 4b).

### 2.5. 3-Dimensional Culture of Patient Derived Mesenspheres

Following BM-MSC characterization of the BM001, BM002, and BM004 patient derived cells, subcultures from each sample were collected and then further cultured in a 96-well aqueous droplet culture platform (3-D Hanging Drop Plate, Perfecta). This platform allowed for the sub-culture of self-assembling spheres of mesenchymal stem cells (mesenspheres). Mesenspheres have been previously shown to exhibit superior regenerative capacity and are known to produce potent anti-inflammatory cytokines [12]. All three of our patient derived mesenchymal cell lines displayed rapid self 3D-assembly within 24 h.

**Figure 5:**
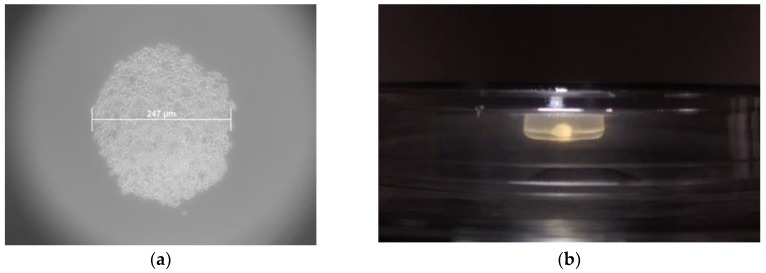
Phase contrast microscope images of hBM-MSCs mesenspheres. (**a**) Microscope image of a BM001 hBM-MSCs mesensphere (247 µm largest diameter) grown using the 96-well Perfecta 3D drop plate (100× magnification); (**b**) Image of a BM001 hBM-MSCs mesensphere (4.6 mm largest diameter) grown using the in-house drop-well device.

MSC suspensions from each patient sample were seeded at a concentration of 2 × 10^4^ cells per hanging drop well and were incubated for 24 h at 37 °C and 5% CO_2_. Sphere forming capacity was assessed in 12 replica wells for each of the three populations studied. Figure 5a demonstrates a mesensphere grown using the BM001 primary cells imaged 24 h after seeding at 10× magnification.

Average sphere size after 24 h was very consistent between the replicas across the sample populations. The diameter of mesenspheres ranged from 212 µm to 253 µm in greatest dimension. All samples tested displayed excellent self-assembly properties indicative of the viable MSC phenotype.

In addition to culturing mesenspheres in the 3D-Perfecta Hanging Drop Plate, an in-house device (drop-well) was fabricated that allowed for the culture of much larger mesenspheres. The in-house device, which is made from a cap of a 1.5 mL microcentrifuge tube glued to a 10 cm tissue culture lid, enabled the cultured spheroids to reach sizes up to of 4.6 mm. These larger self-aggregated stem-cell colonies, not only demonstrated similar internal heterogeneity as their smaller counterparts grown in the Perfecta drop plates, but could theoretically be shaped and re-implanted as is at the site of injury without the need for additional scaffolding material, due to their large size. Figure 5b shows the in-house device with a large active stem-cell culture.

### 2.6. Mesensphere Internal Heterogeneity and Cartilaginous/Calcium Deposition

**Figure 6:**
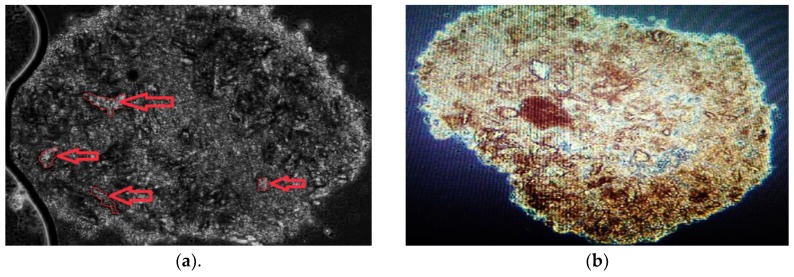
Microscope image of Trypan blue and alizarin red stained mesenspheres. (**a**) Representative microscope image of a cross-section of BM001 hBM-MSCs mesenspheres stained with Trypan Blue. Trypan-Blue stain determined internal cell viability of the spheres, but served also to clearly outline the acellular biologic structures present. Arrows point at four prominent crystalline regions, however the spheroid overall is notably saturated with ECM.; (**b**) Representative microscope image of a cross-section of BM001 hBM-MSCs mesenspheres stained with Alizarin Red showing extensive micro-calcification deposits throughout the sectioned mesenspheres.

After a culture period of 7-days with intermittent media changes occurring every other day, the mesenspheres were fixed, sectioned, and analyzed. It was immediately apparent upon sectioning that a fibrous and mineralized extra-cellular matrix (ECM) had been produced by the self-aggregating stem-cell populations. The ECM was observed in the spheroids from each of the three patient-derived mesenspheres. After sectioning, the spheres were stained and analyzed in order to determine the nature of the observed ECM. Trypan-Blue stain was used in part to determine internal cell viability of the spheres; it also served to clearly outline the acellular biologic structures present. A cross-section image taken of one of the spheres following Trypan Blue staining can be seen in Figure 6a. Arrows point at four prominent crystalline regions, however the spheroid overall is notably saturated with ECM. Alizarin Red stain was also used in order to determine if the deposited ECM was calcified. Figure 6b shows extensive calcification throughout sectioned mesenspheres. Trypan Blue exclusion results demonstrated that the crystalline deposits we imaged were not live cells, but were instead structures resulting from cellular secretions; while the Alizarin Red demonstrated extensive micro-calcification deposits throughout the mesenspheres.

Each of the scaffolds tested in this study was inoculated with a one milliliter suspension consisting of 5 × 10^4^ viable patient derived MSCs. The scaffold was inoculated by directly pipetting the cell suspension into the pores of the scaffold followed by a one-hour incubation period at 37 °C to allow for initial adherence of the cell to the scaffold. Then the scaffold was carefully flooded with culture medium and cultured submerged for a period of 15-days. Following the culture period, the scaffolds were stained and assessed for cell integration and viability.

### 2.7. BM-MSC integration and Viability in Various Man-Made Scaffolds Tested

**Figure 7:**
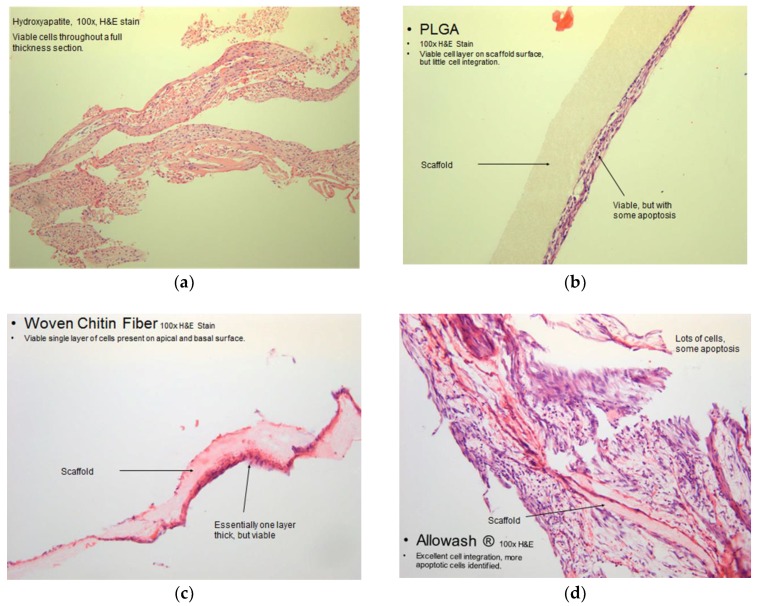
Microscope image of Haematoxylin and Eosin stained scaffolds following culture with hBM-MSCs. (**a**) Representative microscope image of a cross-section of hBM-MSCs seeded and cultured on a 60/40 hydroxyapatite scaffold, and stained with Haematoxylin and Eosin (100× magnification). The viable hBM-MSC population can be seen in significant numbers throughout a full thickness section of the scaffold and the cells do not display prominent apoptotic regions.; (**b**) Representative microscope image of a cross-section of hBM-MSCs seeded and cultured on a PLGA scaffold with 200 µm pores, stained with Haematoxylin and Eosin (100× magnification). The PLGA scaffold did not allow for significant hBM-MSC integration. The MSCs adhered to the outer surfaces of the scaffold and did not migrate far into its interior regions; (**c**) Representative microscope image of a cross-section of hBM-MSCs seeded and cultured on woven chitin fiber material stained with Haematoxylin and Eosin (100× magnification). Also the woven chitin fiber did not support cell growth to a significant degree, showing a viable single cell layer of hBM-MSC cells on the apical and basal surface; (**d**) Representative microscope image of a cross-section of hBM-MSCs seeded and cultured on Allowash cancellous bone scaffold stained with Haematoxylin and Eosin (100× magnification). The Allowash cancellous bone scaffold demonstrated excellent cell integration and promoted MSC proliferation.

The hydroxyapatite scaffold demonstrated superior cell integration and viability properties (Figure 7a) as compared to the other synthetic materials that were tested in this study (Figure 7c,d). Viable MSC population can be seen in significant numbers throughout a full thickness section of the HA scaffold and the cells do not display prominent apoptotic regions (Figure 7a).

Although the PLGA scaffold had superior pliability to the other synthetic materials screened, it did not allow for significant cell integration. The MSCs adhered to the outer surfaces of the scaffold and did not migrate far into its interior regions (Figure 7b).

The woven chitin fiber material did not support cell growth to a significant degree. One fragmented layer of viable cells was identified along its surface (Figure 7c).

The Allowash cancellous bone scaffold demonstrated good cell integration and promoted MSC proliferation. Some apoptotic regions were identified, however this may be a result of the decalcification procedure needed (Figure 7d).

### 2.8. BM-MSC Integration and Viability in Bone Autograft Composites

**Figure 8:**
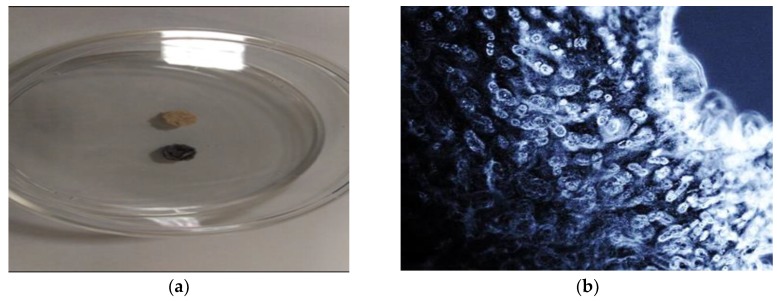
Images of Tetrazolium salt staining of autograft bone composite following culture with hBM-MSCs. (**a**) Image of an in-house autograft bone composite disks stained with tetrazolium salt to assess the viability of the cells. A negative control disk, which did not have cells seeded, was used as a control; (**b**) Inverted light microscopy image of a section from the same scaffold post tetrazolium salt staining (150× magnification).

The in-house autograft bone composite disks were not cohesive enough to withstand the decalcification process and subsequent sectioning on a microtome in order to perform haematoxylin and eosin staining as with the rest of the bio matrices. In order to assess cell viability and integration of the composite disks, the disks were stained with tetrazolium salt to assess the viability of the cells, which resulted in a purple color change if viable cells were present under light microscopy. Figure 8a shows the inoculated scaffold which turned blue for the presence of viable cells and the corresponding negative control following a tetrazolium salt viability staining. Figure 8b shows an inverted light microscopy image of a section from the same scaffold post tetrazolium salt staining at 150× magnification.

## 3. Discussion

As previously reported, several clinical benchmarks need to be achieved in order to develop a successful and scalable MSC based therapy for regenerative medicine. We focused on developing a protocol scientifically rigorous and clinically relevant by outlining a reliable and efficient methodology for obtaining and isolating viable populations of hBM-MSCs to use in a clinical capacity.

Our methodology allows for the expansion of hBM-MSCs in a relatively short amount of time. The protocol we developed cultures MSCs in vitro within a 3-week window. We combined two methodologies: A traditional, two-dimensional substrate-based adherent monolayer method and a three-dimensional spheroid culture method. Notably, the 3D spheroid culture allowed production of hBM mesenchymal cells that retained better osteoblast differentiation capacity over a monolayer culture of hBM-MSCs without the need to use chemical or hormonal modulation.

More importantly, we have demonstrated in this study that the MSCs that were harvested and expanded from trauma donors following a surgical repair of a long bone fracture, maintained their stem-like phenotype, which is necessary for effective wound healing use and is readily adaptable to simple percutaneous harvesting techniques such as iliac crest or trochanteric percutaneous needle aspiration.

Our protocol is uniquely tailored to clinical use due to the utilization of patient derived growth factors present in patient-derived serum (PDS), again obtained with the needle aspirations or serum, instead of animal-derived serum such as fetal bovine serum. In regenerative medicine the use of stem-cell therapies can offer very favorable results, however, in many cases these therapies also pose some major risks. Although fetal bovine serum (FBS) is effective, relatively inexpensive, and easily accessible, its use in cell-based therapies intended for implantation into humans is not an acceptable practice. This is due to the increased risk of pathogen transfer that its use entails. The results of the 3D expansion show the readiness of the newly differentiated osteoblasts to generate new bone and thus potentially initiate the remodeling mechanisms critical for bone healing. We believe that this alone may represent an advancement in atrophic non-union treatment via similar bone grafting harvesting and insertion through percutaneous techniques.

We envision that the integration of this technology in a clinical setting would be a staged process. By developing this method, it is our goal to have patients fulfilling the appropriate criteria present to a referral center to undergo minimally invasive stem cell harvest and expansion ahead of their surgery, and subsequent implantation treatment. Eventually, a referral could be offered for the stem cell expansion only and the surgical implantation procedures could be performed at the referring institution. One even can envision generating custom shaped scaffolds for large bony defects resulting from trauma or tumor resection, possibly even 3D printed, and seeding them with autogenous generated ostoblast stem cells for improved and faster incorporation taking established methods, such as the Masquelet Technique (a 4–12-week process), to the next level [17].

The timing with which we are able to generate autogenous osteoblasts is on par, if not faster than, this membrane based defect auto-grafting methodology commonly employed for bony defects, and potentially could exceed the size restrictions currently limiting this technique.

Future research in this field utilizing the same or similar platforms could yield complimentary regenerative techniques for cartilage, muscle, tendon, skin, and other individual ex-vivo fabrication of autologous patient tissues suitable to repair maxillofacial and orthopedic defects. More studies will be needed to advance this dual culture method based on a traditional, two-dimensional adherent monolayer method and a three-dimensional spheroid culture method to translate it to the clinics.

## 4. Materials and Methods

### 4.1. Study Design and Surgical Procedure for Harvesting hBM-MSCs

Our goal was to standardize the collection and expansion of human bone marrow (hBM) stem cells harvested from patients undergoing intramedullary nail fixation for closed femoral fractures at Marshall University. Patients were selected based on their injuries and suitability for signature of an informed consent in the perioperative setting and enrollment in an IRB protocol at Marshall University (IRB protocol# 393960). The study was conducted in accordance with the ethical principles provided by the Declaration of Helsinki and the principles of good clinical practice.

The hBM samples were collected using a reamer irrigator aspirator (RIA) and filtration system for sample collection and concentration or a standard reaming technique from the fractured femur during the process of preparation of the canal for intramedullary implants. Aspirated material and collected bony fragments from the reaming were transported to the lab within three hours, after which the material was immediately disassociated and plated into a 15cm tissue culture petri dish in RPMI-1640 and incubated at 37 °C in the presence of 5% CO_2_.

### 4.2. Patient Samples

BM001, BM002, and BM004 samples were collected as a bone marrow aspirate from an 11-year-old male, a 25-years old male and a 30-years old female, respectively. Samples were transferred from surgery directly to the tissue culture lab and maintained at room temperature (transfer time was less than 3 h), ensuring optimal sample viability. BM003 was collected as a bone marrow aspirate from a 30-year-old female, but was excluded from the study due to non-viability of the sample.

### 4.3. Patient-Derived Serum Preparation (PDS)

Excess whole blood collected from the surgical site was centrifuged to separate out the various blood components. Blood serum was removed from the platelet layer and filtered through a 0.45 μm membrane. The platelet layer was then lysed with red cell lysis buffer, a hypotonic saline solution, and the resulting suspension was processed through a 0.45 μm membrane. The processed suspension was added back to the whole patient serum. 500 µL aliquots were then frozen and stored at −20 °C until further use. This autologous serum, herein referred to as PDS was added to the basal stem-cell culture media.

### 4.4. Cell Lines and Growth Medium

Osteoblasts (HOB) (PromoCell, catalogue C-12720) were grown in Osteoblast Basal Medium (PromoCell, #C27001). hBM-MSC were plated in RPMI-1640 and later on grown in DMEM-high glucose with Pyruvate (Thermo Fisher Scientific) supplemented with 10% patient-derived serum (PDS) and 2% penicillin/streptomycin solution.

### 4.5. hBM-MSCs Culture and Colony Expansion

After a period of 48 h, the MSCs were transitioned from RPMI-1640 to DMEM media. Cells were then expanded from their original colonies to form confluent monolayers on several 15 cm polystyrene tissue culture treated petri dishes. Accutase^®^ (ThermoFisher Scientifics), was used to sub-culture the hBM-MSCs. In this growth period of approximately two weeks, the cells were monitored on a daily basis for morphological changes and media were replaced twice a week on average. During this phase representative samples were assessed for cell surface markers to further assess if mesenchymal stem cells had been isolated. The hBM-MSCs were then plated in Perfecta 3D Hanging Drop Plates^®^. Cells were seeded in 50 µL drops of DMEM high-glucose medium with L-glutamine and sodium pyruvate and allowed to self-assemble for 24 h prior to viewing. After 24 h the assembled mesenchymal spheres (mesenspheres) were photographed. Mesenspheres were cultured in suspension for 7 days with daily media changes and monitored for circumferential growth. After the 7-day period representative spheres were harvested and analyzed for extracellular matrix (ECM) production and calcium deposition, as well as internal core viability.

### 4.6. Cell Counting

Cells were dissociated using Accutase^®^ (ThermoFisher Scientific) and counted using Trypan blue exclusion method and a Cellometer-Mini Automatic Cell Counter (Nexcelom).

### 4.7. Flow Cytometry Characterization

Cells to be assayed were dissociated using Accutase^®^ (Thermo Fisher Scientific). Incubation with FCR blocking reagent (Miltenyi Biotech) for 15 min was used to limit unspecific antibody binding. Samples were incubated with fluorescence labeled specific antibodies or nothing as negative controls for 30 min at 4oC. Following incubation, cells were washed with flow cytometer buffer and centrifuged to remove the unbound antibodies. Cells were then resuspended in flow cytometer buffer and analyzed through an Accuri C6 Flow Cytometer (BD Biosciences).

### 4.8. Scaffold Preparation and Inoculation with Expanded hBM-MSCs

Various scaffold materials were analyzed for cell integration of hBM-MSCs. Biomaterials routinely used in bone grafting were either purchased or fabricated in house. The use of scaffolding in this study is driven from the clinical desire to fill large defects with an autogenous type bone graft, where typical graft harvesting, such as iliac crest bone graft (ICBG), fails to yield adequate amounts of material for larger voids and thus must be augmented with so-called “bone void fillers”. Furthermore, ICBG harvesting is generally considered to be a very unpleasant, painful and even invasive procedure for the patient. Thus we employed various commonly used and potentially useful candidate materials for study and control purposes while allowing for the development of a percutaneous method of harvesting such as needle aspiration from the iliac wing or even the greater trochanter of the hip [18].

We used a biphasic calcium phosphate bone substitute material with a 60/40 Hydroxiapatite (HA)/Tricalcium Phosphate (TCP) ratio blend biomatrix (kindly donated from Caroline J. Wilcock, School of Clinical Dentistry, University of Sheffield, UK), which is taken to represent the commonly used ceramic based HA bone void fillers in orthopaedic non-unions and defects; Acellular bone composite disc molded from bone fragments collected in reaming at sample collection to represent bone autograft material; Cancellous bone cubes (Allowash^®^) allografts (Life Link Tissue Bank, Tampa, FL); PLGA (poly lactic-co-glycolic acid) constructs with 200 µm pore size (Bioengineering Department, University of Naples “Federico II”, Italy); and Woven chitin derived fiber constructs (kindly donated by Inoue Kuzo, FUENCE Limited, Japan).

The 60/40 Hydroxyapatite biomatrix was chemically hydrated for 3 min using 70% ethanol prior to a saline rinse. Patient derived hBM-MSCs were seeded into the 60/40 hydroxyapatite scaffold and cultured at 37 °C and 5% CO2 in untreated tissue culture Petri dishes using RPMI-1640, and 10% PDS, 2% Penicillin/Streptomycin. The medium was changed every 3 days for 15 days. The inoculated scaffolds were then processed and stained for microscopic analysis of integration, expansion, and differentiation.

Acellular bone autograft composites were molded and sterilized using hot ethanol. The resulting disks weighing approximately 300 mg were then freeze-dried prior to seeding with hBM-MSCs. Allowash ^®^ cancellous bone cubes with an average mass of 230mg were rinsed and hydrated with saline. PLGA derived constructs of varying pore sizes were rinsed in saline prior to seeding with patient derived hBM-MSCs. Woven chitin derived fibers were sterilized in an autoclave prior to hydration with saline. Patient derived hBM-MSCs were seeded into the scaffolds and cultured at 37 °C and 5% CO_2_ in untreated tissue culture Petri dishes using high-glucose DMEM, and 10% PDS, 2% Penicillin/Streptomycin. The medium was changed every 3 days for 15 days. The inoculated disk was then processed and stained for microscopic analysis of integration, expansion, and differentiation. Analysis of the woven fibers scaffold was limited to cell integration.

### 4.9. Hormonal Induction of hBM-MSC Osteogenic Differentiation to Confirm Differentiation Potential

Powder-form dexamethasone (Sigma) was dissolved in DMSO and added to the hBM-MSC culture medium at a final concentration of 100 nm (as per the specification of Tenenbaum and Heersche) for 25 days. The medium was changed every other day. At the end of the 25th day, morphology was assessed and recorded in all samples. The cells were analyzed for cell surface and intracellular osteoblast markers to confirm the presence of differentiated osteoblasts.

### 4.10. Staining Protocols to Study Cell-Seeding Efficacy, Cellular Integration, Vitality, and Differentiation

Methyl Thyazolyl Tretrazolium salt staining. To determine cell-seeding efficacy on scaffolds we used a methyl tetrazolium salt (MTS) assay (MP Biomedical Fisher Scientific). The cell-seeded scaffolds and controls (cells seeded on culture plates) (*n* = 3 for all groups) were thoroughly rinsed in PBS and then incubated in 0.5 mg MTS solution in DMEM high glucose supplemented with 10% PDS. Allowash bone cubes and patient derived composite bone disks that were seeded with the MSC cell populations along with their corresponding negative controls were incubated in 10mL MTS solution at 37 °C in a CO_2_ incubator for 5 h. Bone disks and Allowash cubes were then compared to their unseeded negative controls and images were taken for qualitative data analysis.

Haematoxylin and Eosin staining. To study cellular integration within the various scaffolds, specimens were fixed using a 10% neutral buffered formaldehyde fixative (ThermoFisher) solution for 10 h. Samples were then embedded in paraffin and cut with a microtome (Sakura tissue Tec) at 20 µm sections and mounted onto microscope slides. Slides were processed through an automated Haematoxylin and Eosin staining procedure and mounted with a coverslip for viewing and imaging.

Trypan Blue Staining. To study cellular vitality, 1mL of trypan blue solution (Sigma Aldrich) was used to stain the mesenspheres for 10 min before imaging.

Alizarin Red Staining. To study osteoblast differentiation of the bone marrow cells, mesenspheres were “squash-prepped” onto microscope slides, dehydrated with 95% ethanol, followed by 100% acetone for 2 min each. The slides were dipped in 1 g/50 mL Alizarin red (Sigma Aldrich) solution in water for 3 min. Excess die was flicked off. The slides were dipped in 100% acetone for 30 s followed by 30 s of xylene. Coverslips were mounted to the slides for viewing and imaging. Cellular differentiation was imaged by light microscopy and cells containing mineral deposits were identified by bright red by Alizarin Red staining.

### 4.11. Statistical Analysis

Statistical analysis was performed using GraphPad Prism 6 statistical software (Graphpad, Inc., La Jolla, CA, USA). One and two-way analysis of variance (ANOVA) with Tukey or Bonferroni multiple comparisons post-test was used to determine the statistical significance of the differences between experimental groups. p-values of less than 0.05 were considered statistically significant.

## 5. Conclusions

The bone marrow mesenchymal stem cell isolation and culture protocol we have developed will have a positive impact in the field of head tissue and orthopedic regenerative medicine considering its clinical applications in scenarios where conditions of significant bony defects and fracture non-unions occur. A streamlined clinical protocol of autologous host tissue acquisition to regenerate missing bony structure to alleviate pathologic conditions represents a substantial medical advance in several clinical scenarios, including traumatic bone loss, surgical excision of areas of infected bone, or tumor resections.

## Figures and Tables

**Table 1:** Percentage of cell markers expression in cultured hBM-MSC.

Marker	CD-44	CD-29	CD-105	CD-73
BM001	97.10%	97.80%	97.00%	96.00%
BM002	100%	100%	89.30%	94.00%
BM004	95%	93.50%	96.80%	93.50%

**Table 2:** Number of cells and comparative folds increase of cell growth over 72 h.

Hours	FBS BM001	PDS BM001	FBS BM002	PDS BM002	FBS BM004	PDS BM004
**24**	500 cells	500 cells	500 cells	500 cells	500 cells	500 cells
**36**	746 cells	720 cells	850 cells	823 cells	640 cells	723 cells
**48**	933 cells	1300 cells	1403 cells	2396 cells	835 cells	1244 cells
**60**	1480 cells	1753 cells	1836 cells	2840 cells	1500 cells	2247 cells
**72**	2900 cells	4240 cells	3558 cells	3872 cells	2989 cells	4371 cells
**Fold increase from 24–72 h**	5.8	8.4	7.1	7.7	5.9	8.7

**Table 3:** Comparative percent of expression of the studied markers before and after treatment with dexamethasone.

Cell Marker	ALP	CD105	CD44	CD90	Osteopontin	Osteonectin	Osteocalcin	RUNX2
BM001 (−) Dexa	65.2%	24.3%	6.9%	88.5%	0.0%	19.2%	20.8%	11.9%
BM001 (+) Dexa	100%	97%	54.7%	91.4%	42.4%	57.6%	56.1%	26.3%
Control Osteoblast	93.9%	96.8%	27.9%	90.1%	84.7%	59.6%	53.3%	43.9%
